# Demographic, clinical and biochemical correlates of the length of stay for different polarities in Chinese inpatients with bipolar disorder: A real-world study

**DOI:** 10.3389/fnhum.2023.1135403

**Published:** 2023-03-01

**Authors:** Wei Wang, Jing Du, Sheng Li, Gaoming Xie, Jinjie Xu, Yanping Ren

**Affiliations:** ^1^Beijing Key Laboratory of Mental Disorders, National Clinical Research Center for Mental Disorders & National Center for Mental Disorders, Beijing Anding Hospital, Capital Medical University, Beijing, China; ^2^Advanced Innovation Center for Human Brain Protection, Capital Medical University, Beijing, China

**Keywords:** bipolar disorder, polarity, length of stay, correlates, China, cross-sectional study

## Abstract

**Introduction:** Many patients with bipolar disorder (BD) need hospitalization, while the number of hospital beds for these patients is limited. Managing the length of stay (LOS) is an effective solution to this issue. Research on LOS and its influencing factors in BD is limited in China. This study aimed to identify the factors relevant to LOS in different polarities in Chinese patients with BD.

**Method:** This was a real-world, cross-sectional study. Data were obtained from the electronic medical record system. Patients admitted to Beijing Anding Hospital between Jan 2014 and Dec 2017 and diagnosed with BD were included. Demographic information, clinical characteristics, and biochemical variables were collected. Patients were classified into short and long LOS groups based on a cutoff value. A univariate study and a multivariate logistic regression analysis were performed to identify variables related to LOS in various BD polarities. The receiver operating characteristic (ROC) analysis was utilized to evaluate the discrimination accuracy of the regression model.

**Result:** Four thousand six hundred and seventy-five visits from 4,451 individuals were included in the analysis. For the whole sample, unmarried status, psychotic features, and family history of mental disorders were positively associated with long LOS (all *p* < 0.05). There was an additive interaction between a family history of mental disorders and polarities (*p* < 0.05). For manic episodes, unmarried status, psychotic features, and family history of mental disorders were positively associated with long LOS (all *p* < 0.05). For depressive episodes, psychotic features and high-density lipoprotein cholesterol (HDLC) levels were positively associated with long LOS (all *p* < 0.05). For mixed states, unmarried status was positively associated with long LOS, while low-density lipoprotein cholesterol (LDLC) levels were negatively associated with LOS (all *p* < 0.05). The area under the curve (AUC) values for depressive episodes, manic episodes, and mixed states in the combined model were 0.587, 0.553, and 0.619, respectively (all *p* < 0.05).

**Discussion:** The findings suggested that LOS correlates differed by polarity, with marital status, psychotic features, a family history of mental disorders, and lipid levels strongly linked with LOS in patients with BD.

## Introduction

Bipolar disorder (BD) is a prevalent, chronic, and recurrent mental disorder that has become a leading cause of global disease burden (Alonso et al., 2011; Gore et al., 2011; Webb et al., 2014; Schaffer et al., 2015). A significant proportion of patients with BD may present with psychotic features (e.g., hallucinations, delusions) or hazardous behaviors that may harm themselves or others, which result in hospitalization. Despite the benefits of inpatient treatment, a longer length of stay (LOS) may be counterproductive. Longer LOS is frequently associated with more negative outcomes, such as low patient satisfaction (Bird et al., 2020), mental illness stigma, deterioration of social relationships, and threats to the living situation and career opportunities (Gopalakrishna et al., 2015), which may impact patients’ attitudes toward further psychiatric care (Shi, 2019; Błądziński et al., 2020). China has a significant population of people with BD, but psychiatric ward beds are limited (Que et al., 2019). Longer LOS may rationally and efficiently impact healthcare resource allocation and use. Psychiatric hospitalization accounts for a significant share of mental health expenses, and lengthier psychiatric LOS might incur substantial economic consequences (Habermeyer et al., 2018). Therefore, it is crucial to investigate the variables that may lead to extended LOS for patients with BD in China.

Over the past few decades, several studies conducted in Western countries have reported that the LOS for BD varies across countries, 21.0 days for Sweden (Ragazan et al., 2019), 17.3–20.3 days for Austria (Fellinger et al., 2018), 32.6 days for Ethiopia (Addisu et al., 2015), and 41.2 days for England (Jacobs et al., 2015). Age, gender, marital status, age of onset, course of the disease, and suicidal ideation or attempt were often cited as demographic and clinical correlates of LOS (Jacobs et al., 2015; Deng et al., 2018; Fellinger et al., 2018; Ragazan et al., 2019). However, these studies have some limitations. First, LOS is related to sociocultural factors, while the conclusions obtained from European countries may not be applicable in China. Second, the different polarities of BD (manic episodes/depressive episodes/mixed states) are not distinguished, which may affect the outcome of LOS. Third, most earlier research lacked biochemical variables in patients with BD that have been increasingly shown to be associated with the development and prognosis of the disease.

After reviewing the relevant literature, we selected lipid profiles, immunological components, and hormones for biochemical variables. Previous studies have suggested that aberrant lipid metabolism is prevalent in BD (Chung et al., 2007; Kennedy et al., 2021) and that elevated lipid levels are related to smaller brain structure (Kennedy et al., 2021), more severe mood symptoms (Atmaca et al., 2002; Huang et al., 2018) and cognitive dysfunction (Qiu et al., 2022), lower sleep quality (Soreca et al., 2012), more impulsivity (Tatlidil Yaylaci et al., 2014), and increased drug use (Kumar et al., 2017), all of which may impact LOS. Numerous pieces of evidence continuously have suggested that alterations in the immune system, namely the activation of immune cells and the production of inflammatory compounds, may be associated with changes in the central nervous system (CNS) of BD patients (Pinto et al., 2018). Convincing evidence has shown that altered levels of immune and inflammatory factors are associated with disease risk, symptom profile, cognitive function, treatment response, and prognosis, which affects LOS (Hayes et al., 2017; Park et al., 2017; Bulut et al., 2019; Cuomo et al., 2021). The hypothalamic-pituitary-adrenal (HPA) axis, the hypothalamic-pituitary-thyroid (HPT) axis, and the hypothalamic-pituitary-gonadal (HPG) axis are the three major subsystems of the human neuroendocrine system. Hormonal imbalances in these axes alter the serotonin, dopamine, and glutamate systems in brain areas, which may result in mood, behavior, and cognitive alterations (Niu et al., 2019). Previous studies have shown that indices of hypothalamus-pituitary-adrenal (HPA) axis function is associated with the severity of symptoms of BD (Belvederi Murri et al., 2014). Thyroid hormone levels vary among polarities in BD (Zhao et al., 2021) and are associated with response to medication (Bauer and Whybrow, 2021). Hypothyroidism is associated with rapid transitions to depression or mania in some patients (Buoli et al., 2017). In addition, clinical studies have revealed that gonadal hormones are associated with mood episodes and suicide attempts (Sher et al., 2012). It is concluded that biochemical variables of lipid metabolism, neuroimmune, inflammation, and neuroendocrine may be associated with LOS. According to our knowledge, little research has examined the link between biochemical variables and LOS.

This cross-sectional research aimed to evaluate the demographic, clinical, and biochemical determinants of LOS in various polarities among patients with BD in China. This research provided information for optimizing psychiatric care and allocating mental health resources effectively.

## Materials and methods

### Participants and study design

This study was a real-world, cross-sectional study. Data were obtained from Beijing Anding Hospital, Capital Medical University, one of China’s national medical centers for psychiatric disorders. Patients admitted to Beijing Anding Hospital between Jan 2014 and Dec 2017 and diagnosed with BD were included. Patients were between 18 and 60 (including 18 and 60 years old) and of either gender. For further analysis, BD sub-diagnoses were categorized according to polarity into manic episodes (F31.0–F31.2), depressive episodes (F31.3–F31.5), and mixed states (F31.6). ICD-10-diagnoses F31.7–9 (F31.7 BD, now in remission, F31.8 other BD, F31.9 BD, undefined) were excluded from the study because they were insufficiently specific to be classified.

Independent variables included demographic information, clinical characteristics, and biochemical variables. The dependent variable, LOS, was encoded as a binary variable with “short LOS” defined as ≤28 days and “long LOS” as >28 days. Twenty-eight days are selected as the cutoff value for two reasons: (1) the median LOS for this sample was 28 days, and (2) previous studies on factors associated with LOS in major depressive disorder and schizophrenia also used 28 days as a cutoff value (Cheng et al., 2022a, b).

### Measures

#### Demographic information

At the index time of admission, demographic information was obtained, including age, gender (male/female), and marital status (married/unmarried status; unmarried status comprised single, divorced, and widowed).

#### Clinical characteristics

The patients’ clinical characteristics included the age of onset, course of the disease, comorbid psychotic features, family history of mental disorders, and current polarity (manic episodes/depressive episodes/mixed states).

#### Biochemical variables

Biochemical variables were measured the day after admission using a fully automated biochemical analyzer. Lipid profiles include total cholesterol (TC), triglycerides (TG), high-density lipoprotein cholesterol (HDLC), and low-density lipoprotein cholesterol (LDLC). Immune and inflammatory factors include immunoglobulin G (IgG), immunoglobulin A (IgA), immunoglobulin M (IgM), complement C3 (C3), complement C4 (C4), C-reactive protein (CRP), and erythrocyte sedimentation rate (ESR). Hormones include adrenocorticotropic hormone (ACTH), total triiodothyronine (TT3), total thyroxine (TT4), prolactin (PRL), cortisol (COR), testosterone (TES), estradiol (E2), and progesterone (PGN).

### Statistical analysis

SPSS version 26.0 (IBMCorp., New York, United States) was used for data analysis. The Shapiro-Wilk test was employed to determine if the data distribution was normal. All non-normally distributed continuous variables were reported using the median and quartile range. The differences between the two groups were compared using the rank sum test. The Chi-square test was used to compare categorical data expressed as numbers and proportions (%). The screening criterion was set at *p* < 0.1 to avoid omitting significant variables. The correlations of LOS in the whole sample were investigated using multivariate logistic regression. To determine if the influence of each variable on LOS varied by polarity, an interaction study was conducted between polarity and each variable. Then, multivariate logistic regression analysis was done again to investigate characteristics related to LOS in various BD polarities. A Receiver Operator Characteristic Curve (ROC) study was done to evaluate the LOS prediction accuracy of this regression model. *p* < 0.05 was used as the statistical threshold.

## Results

### Description statistics

Four-thousand six-hundred and seventy-five visits from 4,451 unique individuals were included in the analysis (3,531 individuals were hospitalized once, 826 twice, 70 three times, 17 four times, 5 five times, 1 six times, and 1 nine times). The whole sample had the highest percentage of manic episodes (manic episodes: 62.87%, depressive episodes: 23.91%, mixed states: 13.22%).

The median age of the whole sample was relatively young (31 years for manic episodes, 32 years for depressive episodes, and 29 years for mixed states). In manic episodes, the proportions of males and females were roughly equal. In depressive episodes and mixed states, the proportion of women was slightly higher than that of men. The proportion of unmarried status was significantly higher than married status in each polarity.

In terms of clinical features, more than half of the patients in each polarity had an onset in their early adulthood (the median was 22/23 years). The median course of the disease was six years. Approximately 50 percent of individuals with manic or depressive episodes had psychotic features. More than one-third of patients have family members who used to suffer from mental illness. The LOS of the whole group varied substantially between 1 day and 344 days (1–344 days for manic episodes, 2–211 days for depressive episodes, and 2–161 days for mixed states). The whole sample’s median LOS was 28 days (28 days for manic episodes, 29 days for depressive episodes, and 27 days for mixed states). The average LOS for the whole sample was 31 days (31 days for manic episodes, 31 days for depressive episodes, and 29 days for mixed states). See Table 1.

**Table 1:** Demographic information and clinical characteristics of the whole sample and the three polarities.

Variables	Whole sample	Manic episodes	Depressive episodes	Mixed states
*N* (%)	4,675 (100)	2,939 (62.87)	1,118 (23.91)	618 (13.22)
Demographic information				
Age (years)	31 (24, 42)	31 (25, 42)	32 (25, 44)	29 (23, 37)
Gender				
Male	2,269 (48.53)	1,490 (50.70)	519 (46.42)	260 (42.07)
Female	1,184 (50.17)	1,449 (49.30)	599 (53.58)	358 (57.93)
Marital status				
Unmarried	2,568 (54.93)	1,608 (54.71)	592 (52.95)	368 (59.55)
Married	2,107 (45.07)	1,331 (45.29)	526 (47.05)	250 (40.45)
Clinical characteristics				
Age of onset	23 (18, 30)	23 (19, 30)	23 (18, 31)	22 (18, 28)
Course of disease (years)	6 (2, 12)	6 (2, 12)	6 (3, 13)	6 (2, 11)
Psychotic features	2,054 (50.63)	1,565 (53.25)	489 (43.74)	-
Family history of mental disorders	1,534 (32.81)	976 (33.21)	368 (32.92)	190 (30.74)
LOS (days)				
Range	1,344	1,344	2,211	2,161
Median (IQR)	28 (21, 37)	29 (22, 37)	29 (21, 38)	27 (19, 36)

Continuous variables are presented as median (lower quartile, upper quartile). Categorical data are presented as numbers(%). Abbreviations: LOS, length of stay.

### Univariate analysis of LOS in the whole sample and different polarities

The long LOS group had larger proportions of females, unmarried status, an earlier age of onset, psychotic characteristics and a family history of mental disorders, a lower TG level, and higher levels of HDLC and COR than the short LOS group for the whole sample (all *p* < 0.1).

In patients with manic episodes, the long LOS group was younger, had an earlier start and a longer duration of the disease, had a larger percentage of unmarried status, psychotic features, and a family history of mental disorders, and had a lower PRL level than the short LOS group (all *p* < 0.1).

Compared to the short LOS group, individuals with depressive episodes in the long LOS group exhibited a larger percentage of psychotic characteristics and a higher HDLC level (all *p* < 0.1).

Compared to the short LOS group, the long LOS group of patients with mixed states had greater proportions of females, unmarried status, and family history of mental disorders, lower levels of TG and LDLC, and higher levels of HDLC, IgM, and E2 (all *p* < 0.1). See Table 2.

**Table 2:** Univariate analysis of LOS in the whole sample and the three polarities.

Variables	Whole sample				Manic episodes				Depressive episodes				Mixed states			
	Short LOS	Long LOS	Z/χ^2^	*p*-value	Short LOS	Long LOS	Z/χ^2^	*p*-value	Short LOS	Long LOS	Z/χ^2^	*p*-value	Short LOS	Long LOS	Z/χ2	*p*-value
N (%)	2,360 (50.48)	2,315 (49.52)			1,462 (61.95)	1,477 (63.80)			558 (23.64)	560 (24.19)			340 (14.41)	278 (12.01)		
Demographic information																
Age	31 (25,42)	30 (24,42)	−1.208	0.227	32 (25,42)	30 (24,42)	−1.676	0.094	32 (25,44)	31 (25,44)	−0.41	0.682	29 (23,37)	29 (23,36)	−0.741	0.458
Gender			3.203	0.073			1.037	0.308			0.698	0.404			3.836	0.05
Male	1,176 (49.83)	1,093 (47.21)			755 (51.64)	735 (49.76)			266 (47.67)	253 (45.18)			155 (45.59)	105 (37.77)		
Female	1,222 (52.79)	2,406 (51.47)			707 (48.36)	742 (50.24)			292 (52.33)	307 (54.82)			185 (54.41)	173 (62.23)		
Marital status			30.775	<0.001			28.394	<0.001			2.166	0.539			6.487	0.011
Unmarried	1,202 (50.93)	1,366 (59.01)			728 (49.79)	880 (59.58)			287 (51.43)	305 (54.46)			187 (55.00)	181 (65.11)		
Married	1,158 (49.07)	949 (40.99)			734 (50.21)	597 (40.42)			271 (48.57)	255 (45.54)			153 (45.00)	97 (34.89)		
Clinical characteristics																
Age of onset	23 (18,31)	23 (18,29)	−3.095	0.002	24 (19,31)	23 (18,29)	−3.402	0.001	23 (18,31)	23 (18,30)	−0.438	0.662	22 (18,28)	21 (18,27)	−1.101	0.271
Course of disease	6 (2,12)	6 (2,12)	−1.599	0.11	6 (2,12)	6 (2,12)	−1.928	0.054	6 (3,13)	6 (3,13)	−0.354	0.723	6 (2,11)	6 (2,11)	−0.358	0.72
Psychotic features	976 (48.32)	1,078 (52.92)	8.601	0.003	749 (51.23)	816 (55.25)	4.76	0.029	227 (40.68)	262 (46.79)	4.233	0.04				
Family history of mental disorders	714 (30.25)	820 (35.42)	14.152	<0.001	434 (29.68)	542 (36.70)	16.281	<0.001	191 (34.23)	177 (31.61)	0.87	0.351	89 (26.18)	101 (36.33)	7.407	0.006
Biochemical variables																
TC (mmol/L)	4.35 (3.80, 4.97)	4.33 (3.78, 5.00)	−0.693	0.488	4.29 (3.76, 4.91)	4.27 (3.71, 4.94)	−0.517	0.605	4.38 (3.89, 5.07)	4.43 (3.86, 5.14)	−0.558	0.577	4.53 (3.87, 5.20)	4.34 (3.89, 5.05)	−1.373	0.17
TG (mmol/L)	1.07 (0.75, 1.60)	1.04 (0.73, 1.56)	−1.706	0.088	1.02 (0.71, 1.53)	1.01 (0.71, 1.49)	−1.053	0.292	1.16 (0.80, 1.71)	1.14 (0.83, 1.65)	−0.288	0.773	1.17 (0.82, 1.66)	1.03 (0.74, 1.64)	−1.779	0.075
HDLC (mmol/L)	1.17 (0.99, 1.39)	1.20 (1.01, 1.41)	−2.698	0.007	1.18 (0.99, 1.41)	1.20 (1.01, 1.40)	−1.108	0.268	1.16 (0.97, 1.36)	1.19 (0.99, 1.40)	−2.097	0.036	1.17 (0.96, 1.36)	1.21 (1.01, 1.46)	−2.122	0.034
LDLC (mmol/L)	2.57 (2.10, 3.12)	2.53 (2.05, 3.11)	−1.345	0.178	2.52 (2.06, 3.04)	2.47 (2.03, 3.06)	−0.868	0.385	2.60 (2.15, 3.17)	2.65 (2.10, 3.24)	−0.288	0.773	2.77 (2.21, 3.30)	2.57 (2.13, 3.13)	−2.068	0.039
IgG (g/L)	11.10 (9.60, 12.50)	11.10 (9.62, 12.60)	−0.399	0.69	11.05 (9.57, 12.50)	11.00 (9.57, 12.60)	−0.125	0.901	11.20 (9.73, 12.70)	11.30 (9.71, 12.58)	−0.304	0.761	11.10 (9.53, 12.48)	11.30 (9.69, 12.70)	−1.209	0.227
IgA (g/L)	1.97 (1.45, 2.66)	1.96 (1.45, 2.62)	−0.572	0.568	1.97 (1.45, 2.62)	1.93 (1.44, 2.55)	−1.164	0.245	1.94 (1.42, 2.73)	2.04 (1.49, 2.71)	−0.749	0.454	1.94 (1.47, 2.76)	1.98 (1.42, 2.72)	−0.095	0.924
IgM (g/L)	1.01 (0.73, 1.41)	1.04 (0.75, 1.42)	−1.003	0.316	1.01 (0.72, 1.40)	1.00 (0.73, 1.39)	−0.223	0.824	1.01 (0.74, 1.42)	1.07 (0.77, 1.44)	−1.062	0.288	1.02 (0.75, 1.44)	1.15 (0.83, 1.54)	−2.082	0.037
C3 (g/L)	0.91 (0.78, 1.05)	0.91 (0.78, 1.07)	−0.539	0.59	0.91 (0.78, 1.05)	0.92 (0.78, 1.07)	−0.692	0.489	0.91 (0.77, 1.04)	0.91 (0.77, 1.05)	−0.032	0.975	0.92 (0.78, 1.10)	0.91 (0.79, 1.09)	−0.142	0.887
C4 (g/L)	0.20 (0.17, 0.24)	0.20 (0.17, 0.24)	−1.394	0.163	0.20 (0.17, 0.24)	0.21 (0.17, 0.25)	−1.503	0.133	0.20 (0.16, 0.24)	0.19 (0.16, 0.24)	−0.769	0.442	0.20 (0.17, 0.24)	0.20 (0.17, 0.24)	−0.503	0.615
CRP (mg/L)	0.24 (0.17, 0.42)	0.24 (0.17, 0.42)	−0.011	0.991	0.26 (0.18, 0.45)	0.26 (0.18, 0.45)	−0.573	0.567	0.21 (0.16, 0.34)	0.21 (0.15, 0.33)	−0.137	0.891	0.25 (0.17, 0.42)	0.23 (0.16, 0.40)	−1.14	0.254
ESR (mm/h)	3.00 (2.00, 7.00)	3.00 (2.00, 8.00)	−1.09	0.276	3.00 (2.00, 7.00)	4.00 (2.00, 8.00)	−1.111	0.267	3.00 (2.00, 7.00)	3.00 (2.00, 7.00)	−0.389	0.697	3.00 (2.00, 8.00)	4.00 (2.00, 9.00)	−1.164	0.245
ACTH (ng/L)	41.95 (26.90, 64.00)	41.60 (27.10, 65.60)	−0.818	0.414	42.15 (26.40, 63.75)	40.70 (26.05, 64.15)	−0.095	0.925	41.45 (29.48, 63.23)	43.60 (30.93, 66.43)	−1.555	0.12	41.70 (25.03, 64.48)	41.20 (26.28, 67.33)	−0.545	0.586
TT3 (mol/L)	1.44 (1.27, 1.63)	1.44 (1.27, 1.61)	−0.604	0.546	1.47 (1.29, 1.66)	1.46 (1.29, 1.64)	−1.137	0.256	1.37 (1.21, 1.55)	1.39 (1.22, 1.54)	−0.164	0.87	1.44 (1.26, 1.62)	1.45 (1.30, 1.62)	−0.982	0.326
TT4 (mol/L)	83.25 (72.11, 96.34)	83.25 (71.65, 95.73)	−0.438	0.662	85.36 (73.41, 98.15)	85.03 (73.60, 96.98)	−0.427	0.669	79.97 (69.68, 91.73)	79.31 (68.27, 91.36)	−0.803	0.422	82.03 (71.75, 94.22)	82.73 (71.75, 96.72)	−0.73	0.465
PRL (ng/ml)	33.48 (19.50, 58.77)	32.40 (18.13, 58.74)	−1.581	0.114	38.41 (23.54, 67.84)	35.70 (21.03, 62.86)	−3.017	0.003	23.50 (13.50, 45.20)	24.96 (12.73, 46.44)	−0.271	0.787	28.17 (17.07, 48.24)	30.15 (17.45, 57.79)	−1.043	0.297
COR (μg/dl)	17.94 (13.64, 22.61)	18.34 (13.80, 22.90)	−1.677	0.093	18.14 (13.82, 22.69)	18.43 (13.95, 22.91)	−0.837	0.403	18.12 (13.56, 22.72)	18.31 (13.90, 22.84)	−0.819	0.413	16.87 (13.15, 21.59)	17.89 (13.19, 22.99)	−1.423	0.155
TES (ng/dl)	104.10 (36.10, 399.00)	75.20 (36.70, 382.00)	−0.712	0.476	146.75 (36.78, 420.03)	103.80 (39.50, 410.40)	−0.224	0.823	82.55 (36.33, 375.20)	66.30 (34.43, 338.30)	−1.112	0.266	64.85 (34.05, 340.83)	49.15 (32.45, 309.98)	−1.406	0.16
E2 (pg/ml)	37.14 (23.12, 62.55)	37.40 (23.80, 62.90)	−0.527	0.598	37.49 (23.89, 61.51)	38.00 (24.29, 62.88)	−0.632	0.527	36.12 (20.95, 64.55)	34.58 (21.33, 58.90)	−1.049	0.294	36.05 (23.55, 58.88)	41.34 (26.35, 68.11)	−1.681	0.093
PGN (ng/ml)	0.73 (0.46, 1.18)	0.74 (0.47, 1.14)	−0.154	0.878	0.74 (0.48, 1.16)	0.75 (0.48,1.16)	−0.303	0.762	0.70 (0.44, 1.10)	0.69 (0.45, 1.06)	−0.389	0.697	0.75 (0.44, 1.28)	0.80 (0.49, 1.35)	−0.927	0.354

Continuous variables are presented as median (lower quartile, upper quartile). Categorical data are presented as numbers (%). Abbreviations: LOS, length of stay; TC, total cholesterol; TG, triglycerides; HDLC, high-density lipoprotein cholesterol; LDLC, low-density lipoprotein cholesterol; IgG, immunoglobulin G; IgA, immunoglobulin A; IgM, immunoglobulin M; C3, complement C3; C4, complement C4; CRP, C-reactive protein; ESR, erythrocyte sedimentation rate; ACTH, adrenocorticotropic hormone; TT3, total triiodothyronine; TT4, total thyroxine; PRL, prolactin; COR, cortisol; TES, testosterone; E2, estradiol; PGN, progesterone.

### Multivariate analysis of the whole sample

Multiple logistic regression analysis revealed that unmarried status (OR = 1.454, 95%CI = 1.237–1.710, *p* < 0.001), psychotic features (OR = 1.168, 95%CI = 1.008–1.353, *p* = 0.039) and family history of mental disorders (OR = 1.403, 95%CI = 1.201–1.639, *p* < 0.001) were positively associated with long LOS for the whole sample. There was an additive interaction between a family history of mental disorders and polarity with respect to the probability of a long LOS (OR = 0.626, 95%CI = 0.465–0.841, *p =* 0.002). See Table 3.

**Table 3:** Multivariate logistic regression analysis of LOS in the whole sample.

Variables	OR	*p*-value	95%CI
Gender (male as reference)	1.101	0.218	0.945–1.284
Unmarried status (married as reference)	1.454	<0.001	1.237–1.710
Age of onset	0.995	0.281	0.987–1.004
Psychotic features	1.168	0.039	1.008–1.353
Family history of mental disorders	1.403	<0.001	1.201–1.639
TG	0.995	0.908	0.916–1.081
HDLC	1.081	0.558	0.833–1.404
COR	1.005	0.352	0.994–1.016
Polarities	0.676	0.41	0.266–1.716
Family history of mental disorders* Polarities (manic episode as reference)	0.626	0.002	0.465–0.841

Abbreviations: LOS, length of stay; TG, triglycerides; HDLC, high-density lipoprotein cholesterol; COR, cortisol.

### Multivariate analysis of various polarities

**Figure 1:**
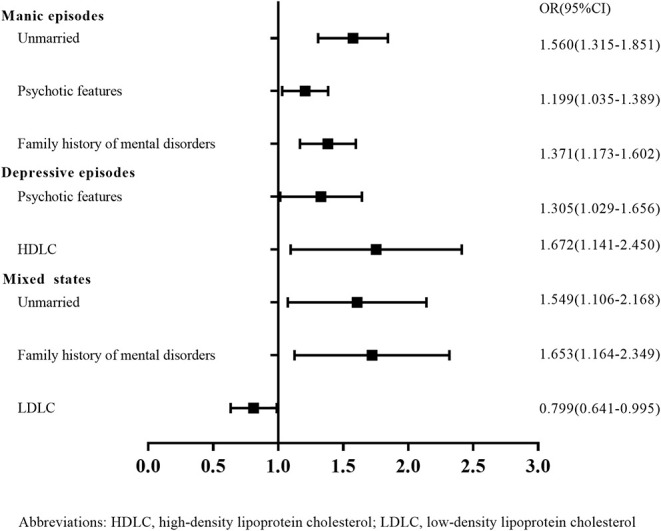
Forest plots depicting logistic regression results of LOS in the three polarities. Abbreviations: HDLC, high-density lipoprotein cholesterol; LDLC, low-density lipoprotein cholesterol; LOC, length of stay.

The results showed that unmarried status (OR = 1.560, 95%CI = 1.315–1.851, *p* < 0.001), psychotic features (OR = 1.199, 95%CI = 1.035–1.389, *p* = 0.015), and family history of mental disorders (OR = 1.371, 95%CI = 1.173–1.602, *p* < 0.001) were positively correlated with protracted LOS in patients with manic episodes. See Table 4 and Figure 1. The AUC of each variable varied between 0.520 and 0.549. The combination model increased diagnostic accuracy with an AUC value of 0.587 (95% CI = 0.566–0.607, *p* < 0.001). See Table 5.

**Table 4:** Multivariable logistic regression analysis of LOS in different polarities.

**Variables**	**Manic episodes**			**Depressive episodes**			**Mixed states**		
	OR	*p-*value	95%CI	OR	*p*-value	95%CI	**OR**	***p*-value**	**95%CI**
Age	1.153	0.728	0.515–2.582	-	-	-	-	-	-
Gender (male as reference)				-	-	-	1.269	0.221	0.86–1.859
Marital status (married as reference)	1.56	<0.001	1.315–1.851	-	-	-	1.549	0.011	1.106–2.168
Age of onset	0.866	0.726	0.387–1.937	-	-	-	-	-	-
Course of disease	0.878	0.752	0.392–1.967	-	-	-	-	-	-
Psychotic features	1.199	0.015	1.035–1.389	1.305	0.028	1.029–1.656	-	-	-
Family history of mental disorders	1.371	<0.001	1.173–1.602	-	-	-	1.653	0.005	1.164–2.349
TG	-	-	-	-	-	-	1.052	0.608	0.867–1.275
HDLC	-	-	-	1.672	0.008	1.141–2.450	1.539	0.153	0.852–2.782
LDLC	-	-	-	-	-	-	0.799	0.045	0.641–0.995
IgM	-	-	-	-	-	-	1.162	0.315	0.867–1.557
PRL	0.998	0.054	0.996–1.000	-	-	-	-	-	-
COR	-	-	-	-	-	-	-	-	-
E2	-	-	-	-	-	-	0.999	0.595	0.996–1.002

Abbreviations: LOS, length of stay; TG, triglycerides; HDLC, high-density lipoprotein cholesterol; LDLC, low-density lipoprotein cholesterol; IgM, immunoglobulin M; PRL, prolactin; COR, cortisol; E2, estradiol.

**Table 5:** Results of ROC curve analysis in different polarities.

Variables	Manic episodes			Depressive episodes			Mixed states		
	AUC	*p*-value	95%CI	AUC	*p*-value	95%CI	AUC	*p*-value	95%CI
Marital status	0.549	<0.001	0.528–0.570	-	-	-	0.551	0.031	0.505–0.596
Psychotic features	0.520	0.059	0.499–0.541	0.531	0.077	0.497–0.564	-	-	-
Family history of mental disorders	0.535	0.001	0.514–0.556	-	-	-	0.551	0.03	0.505–0.596
HDLC	-	-	-	0.536	0.036	0.502–0.570	-	-	-
LDLC	-	-	-	-	-	-	0.547	0.039	0.503–0.594
Combined Model	0.587	<0.001	0.566–0.607	0.553	0.002	0.519–0.586	0.619	<0.001	0.575–0.663

Abbreviations: HDLC, high-density lipoprotein cholesterol; LDLC, low-density lipoprotein cholesterol.

Long LOS was strongly associated with psychotic features (OR = 1.305, 95%CI = 1.029–1.656, *p =* 0.028) and HDLC levels (OR = 1.672, 95%CI = 1.141–2.450, *p =* 0.008) in patients with depressive episodes. See Table 4 and Figure 1. Every variable’s AUC varied between 0.531 and 0.536. The combination model increased diagnostic accuracy with an AUC value of 0.553 (95% CI = 0.519–0.586, *p* = 0.002). See Table 5.

Unmarried status (OR = 1.549, 95%CI = 1.106–2.168, *p =* 0.011) and family history of mental illnesses (OR = 1.653, 95%CI = 1.164–2.349, *p* = 0.005) were strongly linked with protracted LOS in patients with mixed states. A negative correlation was seen between LDLC levels and LOS (OR = 0.799, 95%CI = 0.641–0.995, *p* = 0.045). See Table 4 and Figure 1. Every variable’s AUC ranged between 0.547 and 0.551. The combination model enhanced diagnosis accuracy with an AUC value of 0.619 (95% CI = 0.575–0.663, *p* < 0.001). See Table 5.

## Discussion

According to our knowledge, this is the first study to investigate the demographic, clinical, and biochemical associations with LOS in various polarities among Chinese patients with BD. Our findings indicated that, for the whole sample, unmarried status, psychotic features, and a family history of mental disorders were positively associated with long LOS; however, the interaction between a family history of mental disorders and the polarity was adversely associated with long LOS. Long LOS was positively correlated with unmarried status, psychotic features, and a family history of mental disorders for manic episodes. Long LOS was positively associated with psychotic features and HDLC values for depressed episodes. Long LOS was positively correlated with unmarried status and a family history of mental disorders, whereas LDLC levels were adversely correlated.

The average LOS of Chinese BD patients in this study was 31 days, longer than that of Sweden (Ragazan et al., 2019) and Austria (Fellinger et al., 2018), shorter than that of Ethiopia (Addisu et al., 2015) and England (Jacobs et al., 2015). Possible explanations for the shorter LOS in Sweden and Austria include variations in help-seeking behavior. A greater percentage of identification of emotional and behavioral issues, for instance, might lead to early treatment seeking and encourage quicker recovery (Gaine et al., 2021). This may also be a result of the community-based rehabilitation treatment system for mental illnesses, which allows patients to return to their families and society before all symptoms have disappeared (Kar Ray et al., 2019; Florentin et al., 2021). However, this conclusion may also be affected by a deficiency in medical resources since fewer accessible beds, and higher inpatient demand may result in earlier discharge.

In this study, unmarried status was a risk factor for long LOS for manic episodes and mixed states. Although the results of earlier research on the influence of marriage on LOS are contradictory, more studies have revealed that married patients had a reduced chance of having extended hospital stays than unmarried patients (Masters et al., 2014; Cheng et al., 2022a), which is consistent with our findings. A possible explanation is that marital relationships enhance social networks, providing emotional and financial support. Studies have found that married patients with psychiatric disorders are more resilient than unmarried patients and that resilience is crucial to their recovery (Wingo et al., 2010).

This study showed that psychotic features were a risk factor for long LOS for manic and depressive episodes, which is consistent with previous studies (Tulloch et al., 2011; Dimitri et al., 2018; Ragazan et al., 2019). Ragazan et al. analyzed all inpatients with a first diagnosis of BD in the Swedish population register from 2005 to 2014 and found that psychotic features were a strong predictor of long LOS for manic and depressive episodes (Ragazan et al., 2019). A prospective cohort study across five countries found that psychotic disorder was the strongest predictor of long LOS (Dimitri et al., 2018). A meta-analysis also showed that psychotic features were associated with increased LOS (Tulloch et al., 2011). One possible explanation is that psychotic features may lead to more severe illness and less self-awareness, which results in longer hospital stays. Another explanation is that BD with psychotic features is more likely to be misdiagnosed as schizophrenia-spectrum disorders, and inappropriate treatment may prolong LOS.

This study indicated that a family history of mental disorders was a risk factor for prolonged LOS in manic episodes and mixed states. Similar results were observed in pediatric and adolescent psychiatric disorder inpatients (Zanato et al., 2021). The significant correlation between a family history of mental disorders and hospitalization duration demonstrates genetic susceptibility’s impact. Literature reveals a high relationship between a family history of mental disorders, especially with afflicted parents, and the likelihood of mental disorders and hospitalization in offspring (Reupert et al., 2013; Tossone et al., 2014; Glahn et al., 2019). The presence of mental disorders in more than one family member may lead to poor communication and low family system cohesion, resulting in more intra-family conflict, making it more challenging to stabilize patients in the acute phase and leading to longer LOS (Glahn et al., 2019). Interestingly, our investigation revealed an interaction between a family history of mental disorders and the polarity of BD, such that the impact of a family history of mental disorders on LOS is dependent on polarity.

To our knowledge, this is the first study to evaluate the association between lipid levels and LOS in patients with BD. High HDLC levels were shown to be a contributing factor for prolonged LOS in depressive episodes, whereas high LDLC levels were protective in mixed states. This might be because lipid levels influence the intensity of symptoms and other prognostic variables in BD. Cholesterol is critical for membrane stability and neurotransmission, while disordered cholesterol metabolism may trigger serious central neuroinflammation, leading to abnormal monoaminergic neurotransmission (De Melo et al., 2017; Reponen et al., 2020). Few studies have examined the association between clinical symptoms and lipid levels in BD. Shapiro et al. discovered that among the BD-mixed/hypomanic subgroup, greater mania ratings were related to a higher TG level, a higher TG/HDLC ratio, and a lower HDLC level (Shapiro et al., 2022). Bartoli et al. performed a meta-analysis to investigate variations in lipid profiles between suicide attempters and non-attempters with BD, but they did not find any significant differences (Bartoli et al., 2017). It should be noted that many factors can affect lipid levels and that psychotropic drug use, poor lifestyle, and other factors often exacerbate dyslipidemia in patients with BD. In conclusion, the study of the association between lipid levels, LOS, and clinical characteristics in BD is still preliminary, and further long-term follow-up studies are needed.

## Limitation

Some limitations must still be indicated. Before doing longitudinal investigations, it is difficult to determine causation and directionality from cross-sectional data. Second, lipid levels are affected by several variables, including age, gender, body mass index (BMI), and current psychotropic medication usage, and we cannot rule out the effect of confounding variables. Third, this study did not distinguish between patients with first and recurrent hospitalizations, which could lead to confounding bias. Finally, the AUC of the model in this study ranged from 0.553 to 0.619, which is close to the random model. This may be due to insufficient variables covered in the study, such as quantitative measures of symptom severity, electroconvulsive therapy (Patel et al., 2019), psychotropic medication type and dosage (Deng et al., 2018; Fornaro et al., 2018; Lee et al., 2020), and health insurance (Masciale et al., 2021).

## Conclusion

In conclusion, this study investigated the factors associated with LOS in BD in China. The findings revealed that correlates of LOS varied by polarity, with marital status, psychotic features, family history of mental disorders, and lipid levels significantly associated with LOS. These results emphasize that in the context of inadequate healthcare resources, it is advisable to consider the impact of these critical factors on LOS during the initial patient assessment in order to more appropriately allocate healthcare resources.

## Data availability statement

The raw data supporting the conclusions of this article will be made available by the authors, without undue reservation.

## Ethics statement

The studies involving human participants were reviewed and approved by the institutional review board of the Beijing Anding Hospital, Capital Medical University. Written informed consent for participation was not required for this study in accordance with the national legislation and the institutional requirements.

## Author contributions

YR: study design. WW, JX, and YR: data collection, analysis, and interpretation. WW and JX: drafting of the manuscript. YR, JD, SL, and GX: critical manuscript revision. All co-authors: approval of the final version for publication. All authors contributed to the article and approved the submitted version.

## Abbreviations

BD: Bipolar disorder
LOS: Length of stay
ROC: Receiver operating characteristic
AUC: Area under the curve
HPA: Hypothalamus-pituitary-adrenal
TC: Total cholesterol
TG: Triglycerides
HDLC: High-density lipoprotein cholesterol
LDLC: Low-density lipoprotein cholesterol
IgG: Immunoglobulin G
IgA: Immunoglobulin A
IgM: Immunoglobulin M
C3: Complement C3
C4: Complement C4
CRP: C-reactive protein
ESR: Erythrocyte sedimentation rate
ACTH: Hormones: adrenocorticotropic hormone
TT3: Total triiodothyronine
TT4: Total thyroxine
PRL: Prolactin
COR: Cortisol
TES: Testosterone
E2: Estradiol
PGN: Progesterone
BMI: Body mass index.
